# 278. Effects of polymyxin B direct hemoperfusion by measurement of outer membrane vesicles in patients with septic shock

**DOI:** 10.1093/ofid/ofad500.350

**Published:** 2023-11-27

**Authors:** Kyeong Min Jo

**Affiliations:** Inje university, Haeundae Paik hospital, Busan, Pusan-jikhalsi, Republic of Korea

## Abstract

**Background:**

Sepsis is one of the most common reasons for admission to the intensive care unit, and its incidence continues to increase. However, the precise mechanism of sepsis has not yet been fully elucidated. Outer membrane vesicles (OMVs) present in the outer membrane of bacteria have been speculated to be one of the causes of sepsis. Direct hemoperfusion with polymyxin B-immobilized fiber (PMX-DHP) can be used as a means to remove OMVs. This study evaluated whether the use of PMX-DHP affects bacterial distribution.

**Methods:**

A total of 30 critically ill patients with severe septic shock were enrolled from intensive care unit of Haeundae Paik hospital. Plasma and urine samples were collected before and after the use of PMX-DHP. Sequencing-based metagenome analysis of the microbiome was performed, and changes in the distribution of various bacteria before and after the use of PMX-DHP were identified.

**Results:**

A total of 30 patients were enrolled. Figure 1 shows the changes in the distribution of bacteria in plasma before and after the use of PMX-DHP. The only strain that showed a statistically significant difference in the pre-mean and post-mean values in this distribution was *Bradyrhizobium* (0.0152 →0.0000, p=0.0255, Figure 2). Figure 3 shows the changes in the distribution of bacteria in urine before and after the use of PMX-DHP. No bacteria showed a statistically significant difference in distribution before and after the use of PMX-DHP in this distribution.

Figure 1

**Figure:**
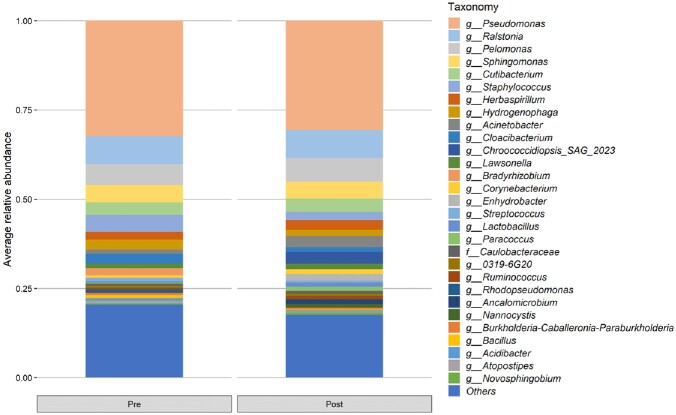

Distribution of bacteria in plasma before and after the use of PMX-DHP

Figure 2

**Figure:**
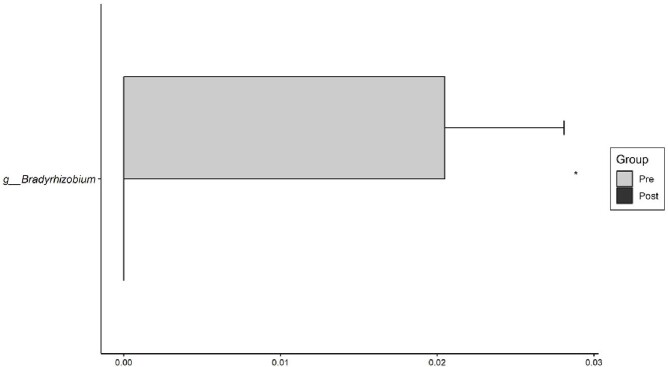

Changes in the distribution of Bradyrhizobium in plasma

Figure 3

**Figure:**
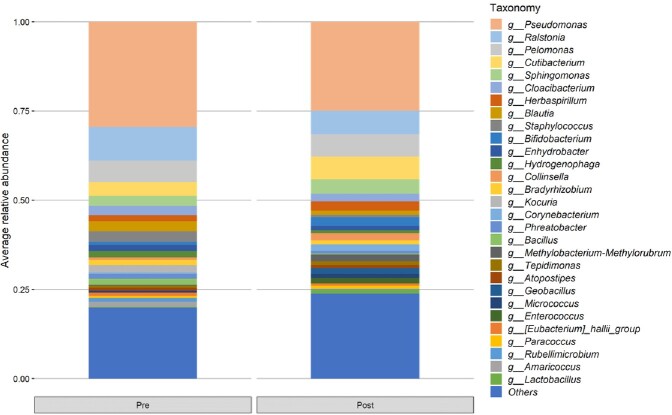

Distribution of bacteria in urine before and after the use of PMX-DHP

**Conclusion:**

*Bradyrhizobium* was the only strain that showed a statistically significant decrease in the amount of outer membrane vesicles (OMVs) in plasma through hemoperfusion using PMX-DHP.

**Disclosures:**

**All Authors**: No reported disclosures

